# Time-Dependent Effects of Pre-Aging 3D Polymer Scaffolds in Cell Culture Medium on Cell Proliferation

**DOI:** 10.3390/jfb3020372

**Published:** 2012-05-22

**Authors:** Kaushik Chatterjee, Stevephen Hung, Girish Kumar, Carl G. Simon

**Affiliations:** 1 Polymers Division, National Institute of Standards & Technology, 100 Bureau Drive, Gaithersburg, MD 20899, USA; Email: kchatterjee@materials.iisc.ernet.in (K.C.); shung1@umd.edu (S.H.); girishkumarsharma@gmail.com (G.K.); 2 Department of Materials Engineering, Indian Institute of Science, Bangalore 560012, India

**Keywords:** 3D scaffolds, biomaterials, osteoblast, protein adsorption, tissue engineering

## Abstract

Protein adsorption is known to direct biological response to biomaterials and is important in determining cellular response in tissue scaffolds. In this study we investigated the effect of the duration of protein adsorption to 3D polymer scaffolds on cell attachment and proliferation. 3D macro-porous polymer scaffolds were pre-aged in serum-containing culture medium for 5 min, 1 d or 7 d prior to seeding osteoblasts. The total amount of protein adsorbed was found to increase with pre-ageing time. Cell attachment and proliferation were measured 1 d and 14 d, respectively, after cell seeding. Osteoblast proliferation, but not attachment, increased with scaffold pre-ageing time and amount of adsorbed serum protein. These results demonstrate that the amount of time that scaffolds are exposed to serum-containing medium can affect cell proliferation and suggest that these effects are mediated by differences in the amount of protein adsorption.

## 1. Introduction

When a tissue scaffold prepared from a biodegradable polymer is placed in a biological fluid such as serum-containing cell culture medium or blood, a number of events occur leading to changes in material properties and ultimately the biological properties to the biomaterial. Protein adsorption is one of the first events that occurs after biomaterial implantation and is critical in determining biomaterial biocompatibility [1]. The process of protein adsorption determines the presentation of functional protein epitopes on the biomaterial surface which strongly influences the outcome of cell-material interactions [2,3]. These processes are complicated by the fact that the amount, species, and conformations of absorbed proteins can vary with a wide range of parameters. The presence of hundreds of serum proteins in cell culture medium and bodily fluids [4] causes competitive protein adsorption events that lead to a dynamic environment at the material-solution interface [1,5,6]. The relative fractions of individual proteins in the adsorbed layer may also change over time due to competitive adsorption between different species [1,5,6].

A number of studies have investigated how surface properties such as chemistry, roughness, and wettability affect protein adsorption to biomaterials and the resultant cellular response. For example, the conformation of adsorbed fibronectin is affected by substrate surface chemistry which affects presentation of integrin binding epitopes and MC3T3-E1 osteoblast adhesion [3]. Another group also demonstrated that attachment of primary human osteoblasts on self-assembled monolayers (SAMs) of different terminal groups is mediated by fibronectin adsorption to the surface, integrin binding and vinculin distribution [7]. When SAM-modified surfaces were introduced to serum proteins, attachment of endothelial cells correlated with the displacement of non-integrin binding-proteins like albumin by integrin-binding proteins like fibronectin and vitronectin [8].

Previous work has also investigated the effect of protein adsorption to 3D tissue scaffolds [9,10]. Fibronectin adsorption in combination with plasma treatment of 3D polymer scaffolds prepared by a precision extrusion deposition technique led to significant increases in cell adhesion and differentiation [9]. In another report, it was observed that the addition of osteoconductive hydroxyapatite to polymer scaffolds enhanced the total amount of adsorbed serum proteins, including the integrin-binding proteins fibronectin and vitronectin [10]. These adsorbed proteins resulted in reduced cell apoptosis in the composite scaffolds relative to cells seeded on scaffolds of the pure polymer [10].

Previously, we demonstrated that pre-ageing time of polymer films in serum-containing medium resulted in variable surface morphology and water-wettability [11]. These changes in polymer surface properties affected osteoblast adhesion and morphology. These results are important since they indicate that the exposure time of flat substrates to serum proteins should be well-controlled during *in vitro* experimentation. The data also suggest that the performance of polymeric biomaterials for use in tissue engineering applications can be improved by pre-aging in serum-containing medium. Since this previous work used 2D polymer films, we focused on 3D scaffolds in the current work since polymers must be fabricated into scaffolds for tissue engineering applications. Scaffolds present varied topographies and cells are sensitive to the topography of their substrate [12,13]. Herein, we tested if time of scaffold pre-aging in serum-containing medium affected osteoblast adhesion and proliferation. 

Many techniques have been developed to fabricate polymeric 3D tissue scaffolds [14,15,16,17]. Herein, porogen-leaching was used since this approach yields scaffolds that support osteogenesis [18]. For porogen-leaching, polymer is dissolved in solvent and mixed with a porogen such as NaCl. Solvent is removed by drying and the porogen is leached in water to generate a macroporous foam scaffold. Poly(ε-caprolactone) (PCL) was the polymer chosen for scaffold fabrication since PCL is a biocompatible polymer that has been used for bone tissue engineering [19]. PCL scaffolds were incubated in serum-containing medium for different pre-aging times and the attachment and proliferation of osteoblasts was measured. The MC3T3-E1 osteoblasts were used to test adhesion and proliferation since they are a well-characterized model for osteoblasts [20,21]. Adhesion and proliferation were measured by fluorescent imaging and DNA quantification after 1 d or 14 d of culture on pre-aged scaffolds.

## 2. Experimental Section

### 2.1. Fabrication of Macroporous Scaffolds

PCL (65,000 g/mol mass averaged relative molecular mass, Sigma) was dissolved in dioxane (Sigma) at 0.1 g/mL. Scaffolds were prepared in 96-well flat-bottom polypropylene plates by adding 30 μL of the PCL solution to each well containing 0.13 g of sieved NaCl crystals (250 μm to 425 μm diameter). Plates were frozen in liquid nitrogen and lyophilized overnight to remove the solvent. Salt from the scaffolds was leached in excess water for 4 d with daily water changes, and subsequently air-dried and stored under vacuum until use. The finished scaffolds were cylindrical, 2.5 mm high and with 6.5 mm diameter. For scanning electron microscopy (SEM), scaffolds were sputter-coated with gold and imaged (15 kV, Hitachi S-4700-II FE-SEM). 

### 2.2. Pre-Ageing and Cell Seeding

MC3T3-E1 mouse cells (Riken Cell Bank, Tsukuba, Japan), a well-characterized osteoblast model, were used for cell culture [20,21]. Cells were cultured at 37 °C in 5% CO_2_ in media prepared from α-modification of Eagle’s minimum essential medium (Invitrogen) supplemented with 10% volume fraction of fetal bovine serum (Gibco) and 0.06 mg/mL of kanamycin sulfate (Sigma-Aldrich), as described previously [22,23]. Passage 3 cells at 80% confluency were used for all experiments. 

Scaffolds in 96-well plates were sterilized in ethylene oxide (Anderson Products) and placed under house vacuum to degas for 3 d. To prepare scaffolds for cell seeding, 0.2 mL medium was added to each well and placed under house vacuum for 2 min to remove air bubbles and wet the pores. For 5 min pre-aged samples, the medium was immediately removed and cells were seeded onto the scaffolds by adding 0.2 mL of medium containing 10^4^ cells such that the scaffolds were exposed to culture medium for 5 min prior to cell seeding. For 1 d or 7 d pre-aged samples, scaffolds in medium were incubated in a cell culture incubator for 1 d or 7 d, respectively, then medium was removed and cells were seeded onto the scaffolds (10^4^ in 0.2 mL of medium). Cells on scaffolds were cultured for either 1 d or 14 d and then stained for fluorescence microscopy or assayed using Picogreen DNA assay. The medium was changed twice per week for 14 d cultures. 

### 2.3. Protein Assay

The amount of protein adsorbed as a result of pre-aging scaffolds in serum-containing medium was measured using the bicinchoninic acid assay kit (BCA kit, Sigma, St. Louis, MO, USA). BCA is a well-established technique for colorimetric quantification of total protein content based on conversion of Cu^2+^ to Cu^+^ by protein and subsequent detection of Cu^+^ by solution containing BCA [24]. After pre-aging scaffolds in medium for 5 min, 1 d or 7 d (in the absence of cells), medium was removed from the scaffolds, replaced with 0.2 mL phosphate buffered saline (PBS) and mixed with a pipet. Scaffolds were washed twice more with PBS and incubated with 0.1 mass% sodium dodecyl sulfate (SDS) in water for 1 h at 37 °C. Aliquots (50 μL) of the SDS solution from scaffolds were mixed with 100 μL of BCA working solution in a fresh 96-well plate. A standard plot was prepared by serial dilution of protein of known concentration. The plate was incubated at 37 °C for 1 h and absorbance was measured at 562 nm using a microplate reader. Seven scaffolds were measured for each pre-aging time (n = 7).

### 2.4. Measuring Cell Response

Cell adhesion and proliferation after 1 d and 14 d of culture was measured by Picogreen DNA assay and fluorescence microscopy. DNA assay was chosen as a quantitative assay and fluorescence microscopy was chosen as a qualitative approach. The DNA content in the scaffold was taken as a measure of the number of cells in the scaffolds since amount of DNA is directly proportional to cell number. DNA was measured using the Picogreen dsDNA Quantitation kit (Molecular Probes), as reported previously [23]. Picogreen reagent is a fluorescent nucleic acid stain for assaying dsDNA in solution [25]. The medium was replaced with 0.2 mL lysis solution containing 0.02% by mass SDS and 0.2 mg/mL Proteinase K for 24 h at 37 °C. Cell lysate (0.1 mL) was transferred to a fresh 96-well plate with 0.1 mL of Picogreen working solution. Fluorescence intensity was measured by a microplate reader using excitation 488 nm and emission 525 nm. A calibration plot prepared from serial dilutions of a known DNA solution was used to calibrate readings.

For nuclear staining and imaging, cells in scaffolds were fixed with 3.7% (mass/volume) formaldehyde in PBS for 15 min at 37 °C, permeabilized in 0.2% by mass Triton X-100 for 5 min at 37 °C, and stained with 1 μmol/L Sytox green (Invitrogen) solution in PBS for 1 h at 37 °C [23]. Stained cells were imaged using an inverted epifluorescence microscope (Nikon Eclipse TE 300) in the green channel. For each condition and time point, 8 scaffolds were used for the DNA assay and 4 scaffolds were used for imaging.

## 3. Results

**Figure 1:**
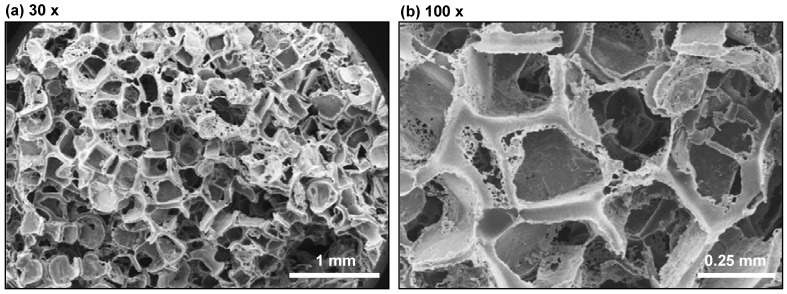
Scanning electron micrographs of 3D poly(ε-caprolactone) (PCL) salt-leached scaffolds at (a) 30× and (b) 100× magnification showing the macroporous structure.

3D PCL scaffolds were prepared by salt-leaching method in 96-well plates. SEM images (Figure 1) present the macroporous foam structure of the scaffold. Large pores with sizes from 0.2 mm to 0.4 mm were present as a result of salt leaching. Porosity of scaffolds was 97% (S.D. = 0.1%) as determined previously by gravimetric methods described previously [26] where scaffold mass was determined by weighing, scaffold volume was determined with calipers and the density of PCL was known (1.1 g/mL).

**Figure 2:**
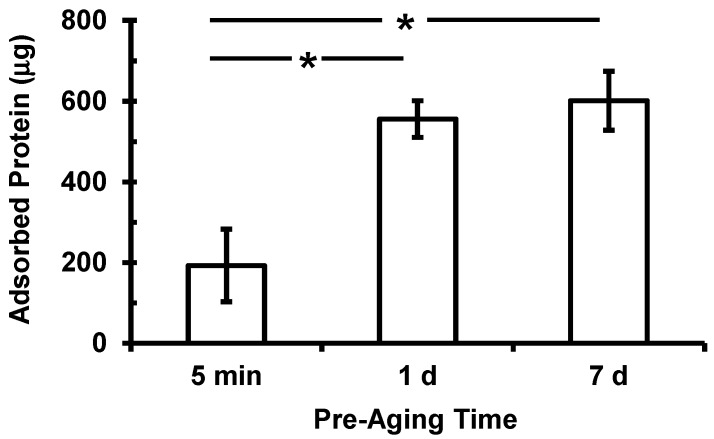
Total amount of protein adsorbed to 3D scaffolds increased with pre-aging time in serum-containing cell culture medium as measured by bicinchoninic acid (BCA) assay. Data are means and error bars are S.D. (n = 7). Statistically-significant differences [analysis of variance (ANOVA) with Tukey’s, *p* < 0.05] are indicated by asterisk.

Prior to seeding cells, scaffolds were pre-aged in serum-containing medium for 5 min, 1 d or 7 d. The total amount of protein adsorbed was found to increase with pre-ageing time (Figure 2). The quantities of proteins adsorbed in scaffolds after 1 d and 7 d of pre-ageing were significantly higher (*p* < 0.05) than in scaffolds pre-aged for 5 min. The amount of adsorbed protein present in scaffolds increased 188% from 5 min pre-aging to 1 d pre-aging and another 8% when comparing 1 d to 7 d pre-ageing.

**Figure 3:**
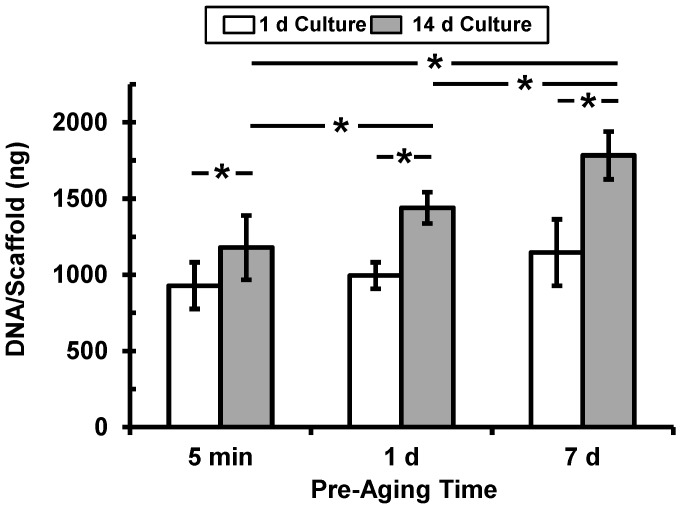
DNA content at 1 d (white bars, cell attachment) and 14 d (gray bars, cell proliferation) after seeding osteoblasts in scaffolds that were pre-aged in serum-containing cell culture medium for 5 min, 1 d or 7 d. DNA was measured by Picogreen DNA assay. Data are means and error bars are S.D. (n = 8). Statistically-significant differences (ANOVA with Tukey’s, p < 0.05) are indicated by asterisk.

DNA content in the scaffolds at 1 d and 14 d after seeding osteoblasts was used as a measure of cell attachment and proliferation, respectively (Figure 3). Osteoblast adhesion at 1 d increased marginally with increasing pre-ageing time (23% increase in DNA from 5 min to 7 d pre-ageing), though this increase was not significant (*p* > 0.05). There was a significant increase (*p* < 0.05) in osteoblast numbers at 14 d (cell proliferation) with increased pre-ageing time. In addition, DNA content at 14 d was significantly (*p* < 0.05) higher than at 1 d in for all pre-ageing times, indicating that osteoblasts proliferated on the scaffolds in all conditions. The ratio of DNA content at 14 d to that at 1 d was found to be 1.3, 1.5 and 2.4 in the 5 min, 1 d and 7 d pre-aged scaffolds, respectively, indicating that osteoblasts proliferated faster in the scaffolds that were pre-aged the longest. These results show that scaffold pre-ageing time did not affect initial osteoblast attachment to 3D scaffolds but did enhance osteoblast proliferation. 

**Figure 4:**
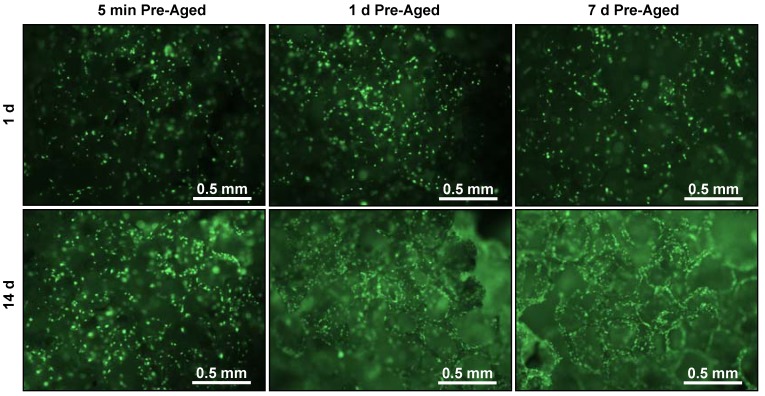
Fluorescent micrographs (40× magnification) of osteoblast nuclei (stained with Sytox green) after 1 d and 14 d culture in scaffolds pre-aged in serum-containing medium for 5 min, 1 d or 7 d.

Fluorescence micrographs of stained osteoblast nuclei (Figure 4) indicate similar cell numbers at 1 d for all three pre-ageing periods. There was an increase in the number of cell nuclei between 1 d and 14 d for all pre-ageing times, but this increase was the greatest in scaffolds pre-aged for 7 d. These qualitative fluorescence microscopy observations corroborate the quantitative results of the DNA assay shown in Figure 3, and suggest that increased scaffold pre-ageing time leads to enhanced osteoblast proliferation without affecting attachment. 

## 4. Discussion

Protein adsorption to biomaterials is a key event that determines biological response to materials. In tissue engineering, serum proteins adsorbed to scaffolds play a critical role in determining the response of cells seeded into the scaffold. Serum contains significant amounts of adhesive, integrin-binding proteins. For example, fetal bovine serum contains 25 μg/mL of fibronectin [27] which contains the RGD integrin-binding peptide sequence [28] that is critical for osteogenesis [29].

Herein, we hypothesized that varying the length of exposure of 3D polymer scaffolds toserum-containing medium would lead to variations in protein adsorption to the scaffolds, and that these variations in protein adsorption would affect cell functions such as adhesion and proliferation. We used PCL, a polymeric biomaterial commonly used to prepare tissue scaffolds [9,23,30]. PCL foams were prepared using NaCl as the porogen to fabricate macroporous structures [23]. We observed an increase in the total amount of protein adsorbed with increased pre-ageing although there was a marginal (statistically insignificant) increase from 1 d to 7 d. These data suggest that a saturating amount of protein is adsorbed on to the scaffold within 1 d of pre-ageing that does not further increase up to 7 d pre-ageing. However, it is possible that the composition (relative molar fraction of individual proteins) of the adsorbed layer and the conformation (availability of functionally-active epitopes) changed from 1 d to 7 d pre-ageing time [5,6]. 

As a model for tissue engineering of bone, we measured the response of an osteoblast cell line. Interestingly, osteoblast attachment at 1 d was not significantly influenced as a result of pre-ageing (Figure 3 and Figure 4). However, increased pre-ageing time resulted in a significant increase in osteoblast proliferation (*p* < 0.05) (Figure 3 and Figure 4). 14 d-DNA content was significantly higher in scaffoldspre-aged for 1 d or 7 d in comparison to 14 d-DNA at 5 min pre-aging (*p* < 0.05) (Figure 3). These results can be explained by the significant increase in amounts of adsorbed proteins between 5 min pre-aging and 1 d or 7 d pre-aging (Figure 2). However, there was significantly more 14 d-DNA content in 7 d pre-aged scaffolds as compared to 1 d pre-aging (Figure 3), even though there was not a significant difference between the amount of adsorbed protein at 1 d and 7 d pre-aging (Figure 2). These results suggest that a change in the total amount of adsorbed protein was not the cause of the increased proliferation for 7 d pre-aging when compared to 1 d pre-aging (Figure 3). The increase in 14 d-DNA content from 1 d to 7 d pre-aging could potentially have been caused by a change in the composition of the adsorbed layer and/or the conformation of the adsorbed proteins [5,6]. Thus, the data suggest that an increase in amount of adsorbed protein caused the increased cell proliferation from 5 min to 1 d pre-aging, but the difference in cell proliferation between 1 d and 7 d pre-ageing could possibly be attributed to changes in the composition and conformation of adsorbed proteins.

The current results agree with the previous observations on 2D films where osteoblast attachment and spreading increased with increased time of pre-ageing in serum-containing cell medium [11]. Taken together, these findings demonstrate that the amount of protein adsorbed to tissue scaffolds can be controlled by varying pre-ageing time in protein-containing medium. Further, this time-dependent variation in the amount of adsorbed protein can be used to control osteoblast proliferation for tissue engineering applications. 

The observation that duration of scaffold exposure to serum can influence cell response has implications both clinically and *in vitro*. Clinically, an implanted scaffold device may be exposed to a patient’s blood well before cells can attach to the scaffold. The amount of time that a scaffold is exposed before cells can colonize the device will depend on many variables such as the type of surgery, the length of the surgery, the composition of the device, the migration speed of the resident cell populations, the expression of adhesion receptors by the resident cells, scaffold porosity, *etc.In vitro*, it is common practice during biological characterization of scaffolds to wet the scaffolds with serum-containing medium prior to cell seeding. The duration of this pre-exposure can vary from minutes to days depending on the lab performing the studies, the goals of the experiment, the materials being used, *etc*. Thus, the results presented here demonstrate that the duration of 3D scaffold exposure to protein-containing medium is an important parameter that can influence cell response and that this parameter should be considered when evaluating scaffold efficacy both *in vivo* and *in vitro*. 

## 5. Conclusions

The total amount of proteins adsorbed to 3D macroporous PCL tissue scaffolds increased with increasing pre-aging time in serum-containing cell culture medium. Increased pre-aging time led to a significant increase in osteoblast proliferation (14 d). These results indicate that osteoblast proliferation in 3D scaffolds can be controlled by varying scaffold pre-aging time and that these effects are mediated by changes in the amount of adsorbed protein. 
